# Challenges faced by professional nurses when implementing the Expanded Programme on Immunisation at rural clinics in Capricorn District, Limpopo

**DOI:** 10.4102/phcfm.v8i2.923

**Published:** 2016-05-13

**Authors:** Tebogo M. Mothiba, Flora M. Tladi

**Affiliations:** 1 Department of Nursing Science, University of Limpopo, South Africa

## Abstract

**Background:**

Immunisation is the cornerstone of primary healthcare. Apart from the provision of safe water, immunisation remains the most cost-effective public health intervention currently available. Immunisation prevents infectious conditions that are debilitating, fatal and have the potential to cause huge public health burdens, both financially and socially, in South Africa.

**Aim:**

To determine the challenges faced by professional nurses when implementing the Expanded Programme on Immunisation (EPI) at rural clinics in Capricorn District, Limpopo Province, South Africa.

**Setting:**

The study was conducted in selected primary healthcare clinics of Capricorn District, Limpopo Province.

**Methods:**

A qualitative explorative descriptive contextual research design was used to gather data related to the challenges faced by professional nurses when implementing EPI at rural clinics in Capricorn District.

**Results:**

The findings revealed that professional nurses had knowledge of the programme, but that they experienced several challenges during implementation of EPI that included staff shortages and problems related to maintenance of the vaccines’ potency.

**Conclusions:**

The Department of Health as well as the nursing administration should monitor policies and guidelines, and especially maintenance of a cold chain for vaccines, to ensure that they are practised throughout Limpopo Province. The problem of staff shortages also needs to be addressed so that the EPI can achieve its targeted objectives.

## Introduction

The Expanded Programme on Immunisation (EPI) was launched in 1974 by the World Health Organization (WHO) with the aim of controlling vaccine-preventable diseases, such as diphtheria, pertussis, tetanus, poliomyelitis, measles and pulmonary TB.^1^ It is one of the most powerful and cost-effective public health programmes aimed at improving child survival and reducing child mortality rates.^2^

Vaccines have become available worldwide. EPI-SA was introduced in 1995 in South Africa and initially covered diphtheria, pertussis, tetanus, poliomyelitis, measles and pulmonary TB. The introduction of combination vaccines as well as improved vaccines contributes to a more successful EPI.^1^ Immunisation schedules for developing countries also need to be adapted to suit the epidemiology of the diseases prevalent in these countries. When looking to add vaccines to the EPI, several factors need to be taken into consideration. These include the burden and significance of the disease within the country, safe and effective vaccine availability, the cost-benefit relationship of the vaccine, whether the country can afford to implement the vaccines, and whether they can be incorporated practically into the national EPI programme.^2^ Several milestones have been reached in the history of EPI-SA: in 1995, hepatitis B vaccine was introduced; in 1999, the *Haemophilus influenzae* type b (Hib) vaccine; and around 2009, rotavirus as well as the pneumococcal conjugate vaccines.^1,2^

Wiysonge et al.^2^ further stipulated that challenges identified by the EPI were linked to healthcare workers who had insufficient knowledge of vaccines and immunisation. Anti-immunisation rumours and reluctance amongst parents and health systems, including insufficient financial and human resources, led to a minimum of adherence to immunisation programmes.^2^ Another fact or that affects EPI utilisation is socio-economic demographics; for example, mothers face transport problems in reaching immunisation centres owing to poor road networks, and lack of funds to meet costs such as food whilst at the centre, transport fare, and the nominal fees collected by staff at the centre.^3^ The immunisation strategy chosen by healthcare personnel also affects attendance by mothers and consequently their children; for example, home visits are aimed at bringing services closer to clients, as opposed to the ‘static strategy’ of healthcare personnel being at a place to which clients must travel to utilise the services at the facility.^3^ Mothers’ understanding of the EPI schedule plays a role in the service’s utilisation because mothers must be aware of the date, time and place for the various vaccines. Healthcare personnel are responsible for informing mothers of infants who need to be immunised.

The WHO reported in 2008 that the number of deaths worldwide from vaccine-preventable diseases amongst children under five years old meant that EPI programmes had not achieved their objectives.^3^ Widsanugorn et al.^1^ have shown that knowledge and practices regarding EPI and the cold chain system amongst healthcare workers in many remote areas of the world are inadequate. Improved knowledge could increase vaccination coverage and the efficiency of EPI and cold chain practices.^4^ The cold chain system comprises three major elements: personnel, equipment and procedures. Masud^4^ found that vaccine-preventable disease outbreaks in areas that had high immunisation coverage might have occurred because of primary vaccine failure. It was behind this background that the present study explored professional nurses’ knowledge and practices of EPI.

**Study objectives**

The objectives of the present study were to:

- explore and describe professional nurses’ knowledge and practices of EPI in the Capricorn District of Limpopo Province, South Africa
- recommend strategies for professional nurses to encourage mothers to adhere to EPI.

## Research method and design

A qualitative research method was utilised which focused on the meaning, understanding and practices of professional nurses regarding the implementation of EPI in their context.^5,6^ An exploratory descriptive and contextual research design was used to explore and describe professional nurses’ knowledge and practices of EPI. This approach assisted the researchers to understand the knowledge and practices in the implementation of EPI. The explorative part of the study was achieved by asking one central question of the participant, which was followed by probing questions regarding unclarified areas. The explorative part of the study was achieved by giving participants the opportunity to describe their knowledge and practices of EPI.^6^

### Study site

This study was conducted at the following clinics that were purposively selected on the basis of the total number of patients seen: Makanye, Nobody and Mankweng Clinics in Capricorn District, Limpopo Province, South Africa. These clinics, according to the stated order, have high patient numbers compared with other clinics in the district.

### Population and sampling

The population comprised all 29 professional nurses in the 3 clinics: Makanye had 8, Nobody had 10, and Mankweng had 11 professional nurses who satisfied the criteria to participate in the study. Non-probability purposive sampling was used to select these clinics. Non-probability purposive sampling was used to include professional nurses in the semi-structured interviews until data saturation was reached.^5^ The researchers included only those professional nurses who had worked two years and more in these clinics on the basis that they had thorough knowledge of the issues studied. A total of 18 professional nurses were interviewed until data saturation was reached.^5,7^

The availability of the nurses during the data collection period was considered at each clinic, and the researchers moved between the three clinics during one day.

### Data collection

The researchers used semi-structured interviews, and participants were asked one central question which was followed by set questions on a guide to further probe the issues studied. The central question which was asked of all participants was ‘Can you kindly explain how EPI is implemented in this clinic?’ The participants were given more time to clarify areas that were not clear to the researchers. Probing questions followed after each first response to the central question, so that researchers could clarify areas that were not clear. A voice recorder was used to capture to capture all interviews, and field notes were written to capture non-verbal cues.^8,9^ Interviews were conducted in a counselling room at each clinic, depending on its availability.

### Data analysis

Tesch’s method of qualitative data analysis as cited by Creswell^7^ was used. Transcripts were read and understood by all participants. Similar topics were grouped together, and those with common features were clustered together until the final themes and sub-themes emerged. Themes and their sub-themes were arranged in columns based on their commonality. Four themes and sub-themes emerged from the data analysis (Table 1).

**TABLE 1:** Themes and sub-themes reflecting professional nurses’ knowledge and practices in the EPI.

Themes	Sub-themes
1. Description of the EPI	1.1 Description of EPI and its purpose
2. Experiences of professional nurses regarding implementation of the EPI	2.1 Challenges experienced by professional nurses 2.2 Shortage of vaccines experienced 2.3 Shortage of staff in healthcare facilities 2.4 Non-compliance of mothers with scheduled return dates
3. Maintaining potency of vaccines	3.1 Principles for maintenance of the cold chain

EPI, Expanded Programme on Immunisation.

### Measures to ensure trustworthiness

Four criteria to ensure trustworthiness were adhered to in the present study, namely credibility, transferability, dependability and confirmability. Credibility was ensured by prolonged engagement with the professional nurses for 14 days during the interview sessions. Dependability was ensured by keeping a secure record of the research process that included voice recordings and field notes. Confirmability was ensured by the researchers who submitted field notes and audio tapes to the external coder who performed independent co-coding of the data. A meeting was held with the researchers to agree on codes which were reached independently. Transferability was ensured by providing dense descriptions of the research methodology followed throughout the study.^8^

### Ethical considerations

Ethical clearance was obtained from the University of Limpopo’s Medunsa Research Ethics Committee (MREC); ethical clearance no. MREC/HS/151/2014: UG. Permission to conduct interview sessions in the clinics was obtained from the Limpopo Province Department of Health and the clinic supervisors. Participants signed informed consent before they could participate in interview sessions, after the researchers explained the aim and objectives of the study. Confidentiality and anonymity were maintained throughout the study.

## Results and discussion

Themes and sub-themes that reflect professional nurses’ knowledge and practices of EPI in the selected clinics in the Capricorn District of Limpopo Province are reflected in Table 1. The themes and sub-themes are discussed, and literature presented to support the study findings. Direct excerpts from participants’ narratives are included to confirm the interview sessions conducted by the researchers.

### Theme 1: Description of expanded programme on immunisation

#### Sub-theme 1.1: Description of expanded programme on immunisation and its purpose

The findings revealed that professional nurses were able to describe what EPI is all about. This was confirmed by the participant who said: ‘The EPI is a programme for immunisation of children which is targeting to prevent and eradicate the following diseases: diphtheria, pertussis, tetanus, poliomyelitis, measles, and TB’.

The EPI was launched in 1974 by the WHO with the aim of controlling vaccine-preventable diseases such as diphtheria, pertussis, tetanus, poliomyelitis, measles and TB.^1^ In South Africa, the EPI was initiated by the Department of Health together with the WHO for children from birth until the age of 12 years for vaccination against infectious diseases such as poliomyelitis, tetanus, pertussis (whooping cough) and others.^2^ The programme’s findings were supported by Hagan et al.^10^ who indicated that there was a need to strengthen immunisation programmes including EPI by empowering nurses with information so that they could work towards achieving its purpose.

### Theme 2: Experiences of professional nurses regarding implementation of the expanded programme on immunisation

The EPI is the most important programme for decreasing child morbidity and mortality rates, because it deals with many diseases that are killing children in South Africa, such as measles, poliomyelitis, hepatitis and others. The practices of professional nurses in this regard areimportant. The following sub-themes emerged.

#### Sub-theme 2.1: Challenges experienced by professional nurses

Professional nurses highlighted that they faced challenges in shortages of vaccines and staff, mothers not always bringing their children back to the clinic on the return dates, and shortages of equipment for immunisation such asRoad-to-Health charts. This was confirmed by a participant who said:

‘You know, there are a lot of problems that we are facing related to the implementation of EPI which include the fact that those vaccines are not enough or are not available to us to immunise the children that come here expecting us to immunise them. There is a lot of shortage of staff who are supposed to immunise the children when they come to the facility and mothers wait for too long with their children. The mothers of children who are scheduled for immunisation are not bringing their children as scheduled and these expose children to diseases.’

This statement was also confirmed by Kitenge and Govender^11^ who found that nurses experience shortages that affect their work, especially in providing immunisations to children.

#### Sub-theme 2.2: Shortage of vaccines

In the clinics, professional nurses sometimes experience shortages of vaccines which makes it difficult for them to follow up on children who missed their innoculations.

‘The challenges are shortage of vaccines, sometimes you find that at 10 weeks the mothers come and do not find the vaccines. There are some vaccines like rotavirus that cannot be given after 6 months, thus children miss some of the doses.’

Kennedy^12^ outlined a worst-case scenario that if individuals are not immunised against diseases, this could result in death or disease transmission to other people.

#### Sub-theme 2.3: Staff shortages at healthcare facilities

Health workers are inequitably distributed throughout the world, with severe imbalances between developed and developing countries. The situation is worsened by imbalances within countries that impede nurses in practising what they learned in terms of the EPI. In general, staff are inadequate in rural areas compared with cities.^11^

Professional nurses in Capricorn District highlighted that:

‘We are facing challenges with staff shortages and this is a burden on us as people who are supposed to provide service because in the clinics there are lots of people coming with different conditions and all need must be attended to. As a result of this staff shortage, some children end up being returned home not immunised.’

This statement is similar to those of Kitenge and Govender^11^ who found that nurses complained that a shortage of personnel contributed towards lack of consistent monitoring of the Road to Health chart, which is a tool for monitoring adherence to children’s immunisation schedules.

#### Sub-theme 2.4: Non-compliance of mothers with scheduled return dates

The study revealed that all mothers didnot comply with the instructions given by nurses during EPI implementation, including non-compliance with scheduled return dates, which affects their performance in terms of immunisation coverage. This finding was confirmed by a participant who said:

‘It feels nice as a nurse to do immunisations if mothers respect the return dates, and it is discouraging when mothers sometimes do not bring the children on the recommended dates because this seems like we are not doing our work, emphasising that mothers must respect their return dates.’

Non-compliance might result from poor understanding of immunisation by mothers of children who are supposed to be immunised. It is claimed that most mothers do not know which diseases are prevented by which vaccines, or how many doses of each are needed, and that is the reason why they sometimes miss the scheduled dates. A study conducted by Wright et al.^13^ concurs with the present study’s findings because it revealed that, in South Africa, mothers do not commit themselves to the immunisation schedules of their children, and this was seen as a barrier to eradicating preventable diseases. In Ethopia, in Arba Minch and the Zuria District, the same problem is experienced: it was found that 20.3% of children were partially immunised and 6.5% were not immunised.^14^

### Theme 3: Maintenance of vaccinepotency

#### Sub-theme 3.1: Principles for maintenance of a cold chain

Ourfindings revealed that the cold chain is not well maintained during transportation and storage of vaccines by the people who are supposed to take care of these processes. A participant reported:

‘Nurses need to maintain the cold chain and ensure that the time period and its lifespan have to be maintained until it is given to the patient but it has to be potent within the set time period.’

The cold chain refers to the system comprising all the people, equipment, transport and procedures that are responsible for ensuring that vaccines reaching the persons who must receive them have been maintained at the appropriate specified temperatures. Most vaccines are heat sensitive, but some are destroyed by freezing. The cold chain is therefore crucial in maintaining vaccine potency. Vaccines should be transported, stored and handled in the appropriate manner from the manufacturer to the point of vaccination.

### Limitations

The study was conducted in Capricorn District clinics in the Mankweng area, and the findings do not allow generalisation to other professional nurses and other districts and provinces in South Africa.

### Recommendations

It is suggested that similar studies be conducted in other provinces and clinics with the aim of determining the practices of professional nurses in implementing the EPI. Accordingly, we recommend that:

- the Department of Health must ensure and enhance optimal immunisation effectiveness by continuous training and regular supervision of professional nurses on EPI implementation
- the Department of Health should employ more staff for providing immunisation services to avoid burn-out of nurses currently in clinics
- the cold chain must be maintained when distributing vaccines to clinics to maintain their potency and effectiveness
- mothers should be taught about the Road to Health chart so that they can identify return dates as well as missing dosages of a vaccine.

## Conclusion

The present study explored the challenges faced by professional nurses when implementing EPI at rural clinics in Capricorn District, Limpopo Province, South Africa. Participants were the clinics’ professional nurses who had experience of EPI implementation. The study showed that professional nurses in the clinics had knowledge and that they frequently implemented the EPI correctly as stipulated by its policies and manual. The cold chain system was not maintained, based on the study findings. The workload at clinics was intense because of staff shortages, and there was also a problem of mothers not complying with scheduled return dates for immunising their children.
